# *Lactobacillus plantarum* and *Lactobacillus reuteri* as Functional Feed Additives to Prevent Diarrhoea in Weaned Piglets

**DOI:** 10.3390/ani11061766

**Published:** 2021-06-12

**Authors:** Matteo Dell’Anno, Maria Luisa Callegari, Serena Reggi, Valentina Caprarulo, Carlotta Giromini, Ambra Spalletta, Simona Coranelli, Carlo Angelo Sgoifo Rossi, Luciana Rossi

**Affiliations:** 1 Department of Health, Animal Science and Food Safety “Carlo Cantoni” (VESPA), Università degli Studi di Milano, 26900 Lodi, Italy; serena.reggi@unimi.it (S.R.); carlotta.giromini@unimi.it (C.G.); carlo.sgoifo@unimi.it (C.A.S.R.); luciana.rossi@unimi.it (L.R.); 2 Department for Sustainable Food Process, Università Cattolica del Sacro Cuore, 29122 Piacenza, Italy; marialuisa.callegari@unicatt.it; 3 Department of Molecular and Translational Medicine (DMMT), Università degli Studi di Brescia, 25123 Brescia, Italy; valentina.caprarulo@unibs.it; 4 Biotecnologie B.T. Srl, 06059 Todi, Italy; aspalletta@biotecnologiebt.it (A.S.); scoranelli@biotecnologiebt.it (S.C.)

**Keywords:** *Lactobacillus plantarum*, *Lactobacillus reuteri*, probiotics, lactobacilli, functional nutrition, diarrhoea prevention, intestinal health, weaned pig

## Abstract

**Simple Summary:**

Antimicrobial resistance is an increasing global concern. Effective alternatives that could replace and reduce antimicrobial treatments in farming have therefore become crucial for animal, human and environmental health. In swine farming, weaning is a stressful phase where piglets can develop multifactorial enteric disorders that require antibiotic treatments. Functional nutrition could thus represent a valuable alternative to reduce and tackle antibiotic resistance. This study assesses the effects of *Lactobacillus plantarum* and *Lactobacillus reuteri* on in-feed supplementation in weaned piglets. After weaning, piglets were allotted to four experimental groups fed a basal diet (CTRL) and a basal diet supplemented with 2 × 10^8^ CFU/g of *L. plantarum* (PLA), *L. reuteri* and a combination of the two strains (P+R) for 28 days. Zootechnical performance and diarrhoea occurrence were recorded. Microbiological and serum metabolism analyses of faeces and blood samples were performed. Supplemented groups with lactobacilli showed a lower occurrence of diarrhoea and improved faecal consistency compared to the control. The PLA group registered the lowest diarrhoea frequency during the 28-day experimental period. The results suggest that dietary administration of *L. plantarum* and *L. reuteri* could prevent the occurrence of diarrhoea in weaned piglets.

**Abstract:**

The effects of *Lactobacillus plantarum* and *Lactobacillus reuteri* and their combination were assessed in weaned piglets. Three hundred and fifty weaned piglets (Landrace × Large White), balanced in terms of weight and sex, were randomly allotted to four experimental groups (25 pens, 14 piglets/pen). Piglets were fed a basal control diet (CTRL, six pens) and a treatment diet supplemented with 2 × 10^8^ CFU/g of *L. plantarum* (PLA, 6 pens), 2 × 10^8^ CFU/g *L. reuteri* (REU, six pens) and the combination of both bacterial strains (1 × 10^8^ CFU/g of *L. plantarum* combined with 1 × 10^8^ CFU/g of *L. reuteri*, P+R, 7 pens) for 28 days. Body weight and feed intake were recorded weekly. Diarrhoea occurrence was assessed weekly by the faecal score (0–3; considering diarrhoea ≥ 2). At 0 and 28 days, faecal samples were obtained from four piglets per pen for microbiological analyses and serum samples were collected from two piglets per pen for serum metabolic profiling. Treatments significantly reduced diarrhoea occurrence and decreased the average faecal score (0.94 ± 0.08 CTRL, 0.31 ± 0.08 PLA, 0.45 ± 0.08 REU, 0.27 ± 0.08 P+R; *p* < 0.05). The PLA group registered the lowest number of diarrhoea cases compared to other groups (20 cases CTRL, 5 cases PLA, 8 cases REU, 10 cases P+R; *p* < 0.01). After 28 days, the globulin serum level increased in PLA compared to the other groups (24.91 ± 1.09 g/L CTRL, 28.89 ± 1.03 g/L PLA, 25.91 ± 1.03 g/L REU, 25.31 ± 1.03 g/L P+R; *p* < 0.05). *L. plantarum* and *L. reuteri* could thus be considered as interesting functional additives to prevent diarrhoea occurrence in weaned piglets.

## 1. Introduction

In livestock farming, effective alternatives to antibiotics that are able to promote health and prevent pathologies are urgently required to tackle antibiotic resistance [1,2,3], and replacing and reducing antibiotic treatments is one of the main targets of European policies [4]. This became even more important after the removal from the market of zinc oxide (ZnO) as a veterinary therapeutic treatment [5,6]. This decision was taken due to the observed increase in heavy metal environmental pollution and scientific evidence showing that ZnO co-selects antibiotic-resistant bacteria [7,8]. ZnO has been used widely after the ban on antibiotics as a growth promoter over the last decade [6,9,10,11]. Alternatives to ZnO and antibiotics are thus required particularly during the weaning phase due to the high incidence of enteric disorders and multifactorial diseases such as post-weaning diarrhoea (PWD) [12,13]. The gastroenteric tract (GIT) is a complex environment where the mucosal chemical barrier, immune system, microbiota and epithelium all impact intestinal health [14,15]. Preserving intestinal health decreases the incidence of pathologies, optimises digestive processes and promotes animal performance. There is increased awareness regarding the role of diet, not only as a physiological requirement, but also in the enhancement of animal and human health and in the prevention of specific pathologies [16]. The modulation of intestinal microbiota by dietary approaches, such as the use of feed additives, is one of the most promising strategies to reduce the risk of pathologies in food-producing animals [17,18]. 

Probiotics are functional feed additives defined as “live microorganisms which, when administered in adequate amounts, confer a health benefit on the host” [19]. Their potential mechanisms of action affect the intestinal microbial ecology through the manipulation of microbiota that lower the luminal pH, the competitive inhibition of pathogen strains, the production of bacteriocins with antimicrobial proprieties and the stimulation of the host immune system [20]. Probiotic supplementation in animal diets helps prevent or treat a variety of intestinal disorders, although their mechanisms of actions are not completely known [21]. Lactic acid bacteria include over two-hundred species and subspecies of which *Lactobacillus* sp., *Lactococcus* sp., *Spreptococcus* sp. and *Enterococcus* sp. are used as probiotics for monogastric animals [22].

*Lactobacillus plantarum* is included in the European register of feed additives [8] as a preservative (1; a), silage additive (1; k), microorganism (1; k) and gut flora stabilizer for chickens (4; b). In several in vitro and in vivo studies, some strains of *L. plantarum* demonstrated a protective activity against epithelial intestinal barrier impairment, restoring the function of thigh junctions and reducing paracellular permeability [23,24]. In addition, *L. plantarum* CGMCC 1258 supplemented at 5 × 10^10^ CFU/kg showed its positive effect in weaned piglets challenged with *Escherichia coli* K88, inhibiting diarrhoea and improving zootechnical performance [25]. In parallel, *Lactobacillus reuteri* was included in the EU feed additive register as a microorganism (1; k) until its withdrawal in 2012 [8] due to a lack of the required documentation. This microorganism is not seen as being dangerous and no issues related to its safety were mentioned in the EU commission decision [26], since it is included in the Qualified Presumption of Safety (QPS) list of the European Union [27].

*Lactobacillus reuteri* I5007 has shown a potential to improve thigh junction expression in newborn piglets and has been found to have protective effects after lipo-polysaccharide (LPS)-induced stress in vitro [21]. *L. reuteri* strains TMW1.656 and LTH5794 produce reuteran which can decrease the adhesive capacity of ETEC *E. coli* [28]. However, several studies have shown the positive impact of various *L. plantarum* and *L. reuteri* strains on improving piglet performance, diarrhoea prevention, stress alleviation, immunity and microbiota modulation [29]. 

Since few papers have assessed the effects of *L. plantarum* and *L. reuteri* strains and their synergy through a wide range of bacterial combination and supplementation levels, more studies are required to clarify the functional proprieties and the optimal inclusion level of these two bacterial strains on diarrhoea prevention in weaned piglets. In addition, probiotics may interact with the host metabolism [30] through their hypocholesterolemic and liver protection effects [31,32]. Furthermore, the bacterial combination does not always result in a synergistic effect, also showing possible competition among probiotic strains [33]. The aim of the study was to evaluate *L. plantarum*, *L. reuteri* and whether their combined supplementation reflects synergistic or antagonistic effects on diarrhoea prevention, metabolic status and performance in weaned piglets.

## 2. Materials and Methods

### 2.1. Species-Specific PCR

Single colonies of *L. plantarum* and *L. reuteri,* isolated from swine, obtained from the Biotecnologie BT (Perugia, Italy) strain collection were cultured in De Man, Rogosa and Sharpe (MRS) medium for 24 h in anaerobiosis conditions at 37 °C. Bacterial strains were diluted in 20 µL of lysis solution (microLYSIS^®^ solution, Clent Life Science, Stourbridge, England) and thermically lysed following the manufacturer’s instructions. After lysis, in order to confirm bacterial species, 2 µL of extracted DNA was used for a PCR reaction through species-specific primers, following the protocol previously described by Torriani et al. [34] for *L. plantarum* and Song et al. [35] for *L. reuteri*. PCR reaction was performed with 17 µL of PCR master mix (Client Life Science, Stourbridge, UK) and 0.5 µL (0.25 µM) of specific primers. *L. plantarum* ATCC^®^14917™ and *L. reuteri* DSM 20016 DNA were included as positive controls.

### 2.2. Minimal Inhibitory Concentration (MIC)

In order to assess the possible presence of antibiotic-resistant genes in *L. plantarum* and *L. reuteri* isolated strains, a minimal inhibitory concentration test was performed. MICs were assessed following ISO 10932 IDF 223 guidelines, adopting VetMIC Lact-1 (version 1) and VetMIC Lact-2 (version 2) (National Veterinary Institute, SVA) (Annex I and II). *L. plantarum* ATCC^®^14917™ was included as a positive control. *L. reuteri* and *L. plantarum* were tested for 16 antibiotic molecules (gentamicin, kanamycin, streptomycin, neomycin, tetracycline, erythromycin, clindamycin, chloramphenicol, ampicillin, penicillin, vancomycin, quinupristin/dalfopristin, linezolid, trimethoprim, ciprofloxacin and rifampicin).

### 2.3. Gastric Acid and Simulated In Vitro Digestion Resistance 

Bacterial cultures of *L*. *reuteri* and *L*. *plantarum* were diluted in MRS broth in order to obtain an optical density of 0.1 measured at 600 nm (V-630 UV-vis, Jasco Deutschland GmbH, Germany). For the gastric acid tolerance test, both bacterial strains were incubated at different pH levels (2, 3, 4, 5, 7 and the control, i.e., medium in which the pH had not been changed), obtained by adding HCl (1 M) monitoring with a pH meter. To perform this assay, bacterial cultures were incubated for 1 h at 30 °C. Bacterial cultures were then diluted and plated on MRS agar using the overlay method [36]. Plates were incubated at 30 °C and the colonies were counted after 48 h. Strain tolerances to in vitro-simulated gastrointestinal tract (GIT) conditions were evaluated according to Charteris et al. [37] and Jensen et al. [38], with minor adaptations. Three independent assays were performed for each strain. The MRS broth with lactobacillus inoculum were incubated at 37 °C for 24 h in anaerobic conditions. To simulate the oral phase, a 10-mL bacterial culture aliquot was added to 10 mL of a sterile electrolyte solution (0.22 g/L CaCl_2_, 16.2 g/L NaCl, 2.2 g/L KCl and 1.2 g/L NaHCO_3_) containing 2.0 g/L pepsin (Sigma-Aldrich Co., Saint Louis, MO, USA) and the first sampling was performed. A gastric resistance assay was performed by adjusting the pH to 3.0 by the addition of HCl (1 M) to activate pepsinogen. The sampling was performed after 90 min of incubation at 37 °C under stirring. A total of 2 mL was then sampled in two tubes for each strain and the cell pellets were obtained by centrifugation at 12,000 rpm for 5 min at 4 °C. In order to simulate duodenal shock phase, the bacterial pellet of one tube for each strain was resuspended in 2 mL of sterile saline solution (16.30 g K_2_HPO_4_, 0.9 g KH_2_PO_4_) supplemented with 0.25 g of porcine bile extract (Oxgall, Merck, Darmstadt, Germany) and subsequently sampled after 10 min of incubation at 37 °C. To evaluate lactobacilli resistance to intestinal conditions, the bacterial pellet of the remaining tube of each strain was resuspended in 2 mL of sterile saline solution containing 0.075 g of porcine bile extract and 0.025 g of porcine pancreatin (Sigma-Aldrich Co., Saint Louis, MO, USA). The last sampling was performed after the incubation for 240 min at 37 °C. Bacterial viability was assessed by plate counting on MRS agar for each sampling point using the overlay method [36]. Plates were then incubated at 30 °C for 48 h, and visible colonies were enumerated. 

### 2.4. Small-Scale Fermentation and Freeze-Drying Resistance

In order to optimise biomass production conditions for experimental trial dietary inclusion, a small-scale fermentation was adopted. A 3 L bioreactor was employed to produce bacterial biomass inoculating fresh *L. reuteri* and *L. plantarum* cultures in 2.5 L of MRS (pH 5.2) supplemented with 2% of glucose maintained at 37 °C, stirred at 10 rpm, to harvest bacteria after 18 and 24 h. Bacterial biomasses were weighted after centrifugation at 4 °C, 4800 rpm, for 25 min. Viability was assessed by resuspending bacterial biomass and performing plate counting after 48 h of incubation under anaerobic conditions at 37 °C. Bacterial samples stored at −80 °C were freeze-dried and samples were heated for 1440 min with 0.2 mbar of pressure for the condenser. The biomass obtained and the vitality of lactobacilli strains were measured by weighting and plate counting, respectively.

### 2.5. Bacterial Fermentation for Experimental Trial Batch Production 

Large-scale fermentations were adopted following the previously described conditions. A total of 3 L of fresh bacterial cultures were inoculated to 30 L of MRS (pH 5.2) supplemented with 1% of saccharose, maintained at 35 °C, and stirred at 10 rpm for 24 h. Biomass was harvested through centrifugation at 4200 rpm, 4 °C for 45 min and cryopreservation solution (43 g/L Na citrate, 28.6 g/L glucose, 28.6 g/L saccharose, 28.6 g/L milk powder and 28.6 g/L ascorbic acid; 1:2, *w*/*v*) was added before free-drying.

### 2.6. Experimental Design, Animal Housing and Dietary Treatments

The experimental trial was performed in accordance with European regulations [39] and approved by the Animal Welfare Organisation of University of Milan (OPBA authorisation n° 09/2020). The in vivo trial was performed on a commercial farm free from pathologies included in the ex-list A of World Organization of Animal Health (OIE): atrophic rhinitis, Aujeszky disease, porcine reproductive respiratory syndrome, salmonellosis and transmissible gastroenteritis. Three-hundred and fifty piglets (Landrace × Large White) weaned at 28 ± 2 days and homogeneous in terms of sex (50% male and 50% female) and weight (7.48 ± 1.07 kg) were identified by individual ear tags and randomly divided into four experimental groups. Animals were allotted in 25 different pens (14 piglets/pen) in standardised environmental conditions (27 °C, 60% relative humidity) for 28 days. After three days of an adaptation period when the animals were fed the same basal diet in order to enable them to overcome the typical post weaning fasting, piglets were assigned to four experimental groups and were fed ad libitum: the control group (CTRL: 84 piglets, 6 pens, 7.46 ± 0.13 kg) the basal diet; the *L. plantarum* treated group (PLA: 84 piglets, 6 pens, 7.49 ± 0.12 kg) basal diet supplemented with 2 × 10^8^ CFU/g of *Lactobacillus plantarum*; the *L. reuteri* treated group (REU: 84 piglets, 6 pens, 7.62 ± 0.12 kg) basal diet plus 2 × 10^8^ CFU/g of *L. reuteri,* and the *L. plantarum* and *L. reuteri* combination group (P+R: 98 piglets, 7 pens, 7.36 ± 0.11 kg) fed basal diet plus 1 × 10^8^ CFU/g of both bacterial strains. Treatments were balanced for each group. The P+R group was characterised by one additional pen in order to include the entire trial room in the experimental design. All the diets were isoproteic and isoenergetic (Table 1) balanced using Plurimix System^®^software (Fabermatica, Cremona, Italy) in line with nutritional requirements for post-weaned piglets [40], and were provided by Ferraroni S.p.A. (Cremona, Italy). Considering the small amount of freeze-dried lactobacilli powder included, the bacterial strains were premixed with wheat flour to ensure a homogeneous dispersion before being added to the horizontal mixer. For the whole diet, 2% of the wheat meal was substituted with 2% of the experimental mix (wheat flour + bacterial strain in order to reach a concentration in the final preparation of 2 × 10^8^ CFU/g).

Experimental diets were analysed in duplicate for lactobacilli viability by plate counting and principal nutrient content [41]: dry matter (DM), crude protein (CP), ether extract (EE), crude fiber (CF) and ash concentrations. DM was obtained by drying samples in pre-weighed aluminium jars through a forced air oven at 65 °C. CP was determined by the Kjeldahl method. EE was assessed by performing ether extraction in a Soxtec. CF was determined by the filtering bag method. Ash content was measured after incinerating samples in a muffle furnace at 550 °C. The fatty acid profile of the experimental diets was analysed starting from a total lipid extraction and the fatty acid methyl esters were prepared according to Christie and Han [42]. The fatty acid analysis was carried out using gas chromatography (TRACE GC Ultra, Thermo Fisher Scientific, Rodano, Italy) fitted with an automatic sampler (AI 1300, Thermo Fisher Scientific) and flame ionization detector (FID). An RT-2560 fused silica capillary column (100 m × 0.25 mm × 0.25 μm film thickness; Restek, Milan, Italy) was used with a programmed temperature from 80 °C to 180 °C at 3 °C/min, then from 180 °C to 250 °C at 2.5 °C/min, which was then held for 10 min. The carrier gas was helium at 1.0 mL/min with an inlet pressure of 16.9 psi. A quantitative procedure was used where 1 mL of internal standard (1 mg/mL 23:0 methyl ester; N-23-M; Nu-Chek Prep Inc., Elysian, MN, USA) was added prior to methylation. The fatty acid methyl ester (FAME) contents were quantified by weight as a percentage of the total FAMEs. All analyses were performed in duplicate.

### 2.7. Animal Performance, Diarrhoea Occurrence and Biological Sample Collection

Body weight (BW) was recorded individually at day 0 (T0), day 7 (T1), day 14 (T2), day 21 (T3) and day 28 (T4). Feed intake was recorded weekly by measuring the feed refused for each pen, considering the pen as the experimental unit. Other performance parameters: average daily gain (ADG), average daily feed intake (ADFI) and feed conversion ratio (FCR) were calculated. Four piglets per pen were randomly selected for faecal sample collection and microbiological analysis (24 piglets CTRL, 24 piglets PLA, 24 piglets REU, 28 piglets P+R; balanced per weight and sex) for the entire experimental period.

Diarrhoea occurrence was recorded weekly by evaluating the faecal consistency which was given a faecal score: a four-level scale (0 = dried consistency, 1 = soft consistency, 2 = mild diarrhoea, 3 = severe diarrhoea). Faecal colour was evaluated through a three-level colour scale: 1 = yellowish, 2 = greenish, 3 = brown; considering ≥ 2 as a normal score [12]. 

Blood samples were obtained from the jugular vein at T0 and T4 through vacuum tubes from two randomly selected piglets per pen, balanced in terms of weight and sex, maintained over time for the entire experimental period.

### 2.8. Microbiological and pH Evaluation of Faecal Samples

Faecal samples were analysed for the total countable bacteria, lactic acid bacteria and coliform bacteria through three different types of culture media: Plate Count Agar (PCA), De Man, Rogosa and Sharpe Agar (MRS) and Violet Red Bile Broth Agar (VRBA), respectively. One gram of faecal sample was diluted and homogenised with 10 mL of sterile 0.9% NaCl solution and centrifugated (3000 rpm, 10 min) to collect the supernatants. Samples were then serially diluted tenfold, and microorganisms were enumerated by plate counting after 24 h of incubation at 37 °C. The lactic acid/coliform bacteria ratio was calculated based on plate counting data from MRS and VRBA agar. The results were expressed as log_10_ of colony-forming units per gram of faeces (log_10_ CFU/g). Fresh faecal samples of pH of T4, diluted in 10 mL of 0.9% NaCl solution and subsequently centrifugated, were measured on the supernatant through a pH meter.

### 2.9. Serum Metabolites 

Serum samples were obtained by centrifugation (3000 rpm, 15 min) and analysed for the concentration of: total protein (g/L), albumin (g/L), globulin (g/L), albumin/globulin (A/G ratio), alanine aminotransferase (ALT-GPT; IU/L), glucose (mmol/L), urea (mmol/L), creatinine (µmol/L), total bilirubin (µmol/L), total cholesterol (mmol/L), triglycerides (mmol/L), high-density lipoprotein (HDL; mmol/L), low-density lipoprotein (LDL; mmol/L), phosphorus (mmol/L) and magnesium (mmol/L) levels with a multiparametric autoanalyzer for clinical chemistry (ILab 650; Instrumentation Laboratory Company, Lexington, MA, USA) at 37 °C. Serum concentration of interleukins 3, 6 and 10 were also quantified immunoenzymatically using enzyme-linked immunosorbent assay (ELISA) kits specific for swine species according to the manufacturer’s instructions (Bioassay Technology Laboratory, Shanghai, China), and concentrations were calculated by fitting the relative standard curves with CurveExpert 1.4 software.

### 2.10. Statistical Analysis 

The results were analysed using a repeated-measures ANOVA using JMP 14 Pro^®^ (SAS Inst. Inc., Cary, NC, USA). Zootechnical performance, faecal score data and faecal bacterial counts were evaluated using a full factorial model (Treatment: Trt, Time: Time, Interaction: Trt × Time). Data related to acid resistance, in vitro simulated digestion, blood metabolism and faecal pH at T4 were assessed through analysis of variance (ANOVA). Diarrhoea incidence was obtained by converting the faecal score data into a dichotomous variable (presence or absence) in order to evaluate observed frequencies through the Pearson’s Chi-Squared test. Multiple comparisons among groups were evaluated by performing Tukey’s Honest Significance Difference test (Tukey’s HSD). The results were presented as least square means ± standard errors (SE). The means were considered different when *p* ≤ 0.05 and statistically tendent for 0.09 ≤ *p* < 0.05.

## 3. Results 

### 3.1. Species-Specific PCR

PCR reaction confirmed the expected fragment of 318 bp for *L. plantarum* and 303 bp for *L. reuteri* (Figures S1 and S2).

### 3.2. Minimal Inhibitory Concentrations

The results of the MIC concentrations tested revealed a bacterial susceptibility to a wide range of antibiotics (Table S1).

### 3.3. Acid and Simulated In Vitro Digestion Resistance 

**Figure 1:**
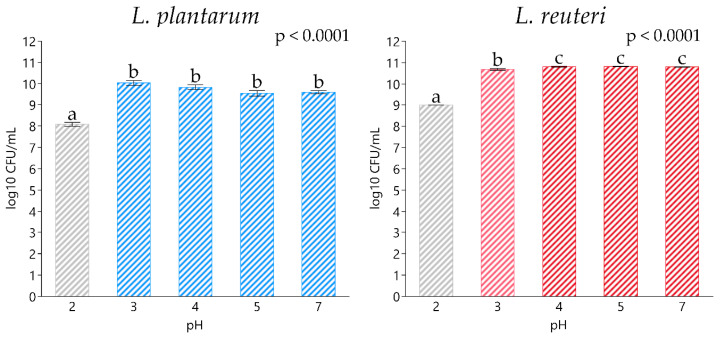
Acid resistance assay from pH 2 to 7 for *L. plantarum* and *L. reuteri*. Data are expressed as least square means (LSMEANS) and standard errors (SE). ^a,b,c^ Means with different superscript letters indicate statistically significant differences (*p* < 0.05).

**Figure 2:**
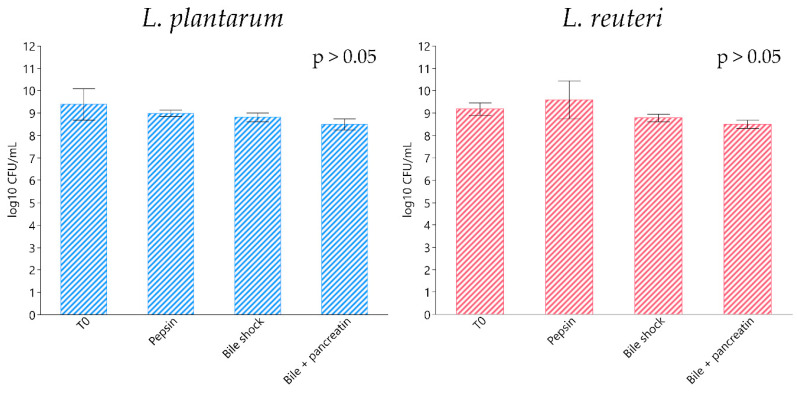
Simulated in vitro gastrointestinal digestion resistance of *L. plantarum* and *L. reuteri.* Data are expressed as least square means (LSMEANS) and standard errors (SE). T0 corresponds to the initial microbial charge measured before gastrointestinal environment simulation.

Bacterial strains exposed to a different pH range showed a statistically significant drop in viability at pH 2, with *L. plantarum* and *L. reuteri* registering a bacterial count of 8.09 ± 0.11 and 9.00 ± 0.02 log_10_ CFU/mL, respectively (*p* < 0.0001), compared to their relative controls at pH 7 (9.60 ± 0.08 and 10.79 ± 0.02 log_10_ CFU/mL, respectively) (Figure 1). Regarding the simulated gastrointestinal digestion, both bacterial strains exhibited an optimal capacity to survive with each tested condition, including gastric juice, bile shock and intestinal juice, without registering any significant decrease in viability compared to their relative initial microbial charge (Figure 2).

### 3.4. Small-Scale Fermentation and Freeze-Drying Resistance

The results of small-scale fermentations showed similar biomass gain and CFU/g viability for both strains considering 18 and 24 h of fermentation time and registering 2.25 × 10^11^ CFU/g biomass for *L. plantarum* and 1.72 × 10^11^ CFU/g biomass for *L. reuteri* after 24 h of fermentation (Table S2). Freeze-drying led to a loss in viability of about 1 log considering the initial lactobacilli count (Table S3).

### 3.5. Bacterial Fermentation for Experimental Trial Batch Production 

Large-scale fermentation performed with 30 L of bacterial culture produced 206.15 and 376.43 g of *L. plantarum* and *L. reuteri* biomass, respectively (Table S4). The freeze-dried bacteria were then used to prepare the experimental diets for a final concentration in feed of 2 × 10^8^ CFU/g.

### 3.6. Evaluation of Experimental Diets

Experimental diet evaluation of lactobacilli viability and principal nutrient content revealed a bacterial viability loss of 10% and nutrient concentrations in line with NRC [40] guidelines, thus fulfilling the nutritional requirements of weaned piglets. The inclusion of bacterial strains did not influence the nutrient profile of treatment groups (Table 2).

### 3.7. Zootechnical Performance 

**Figure 3:**
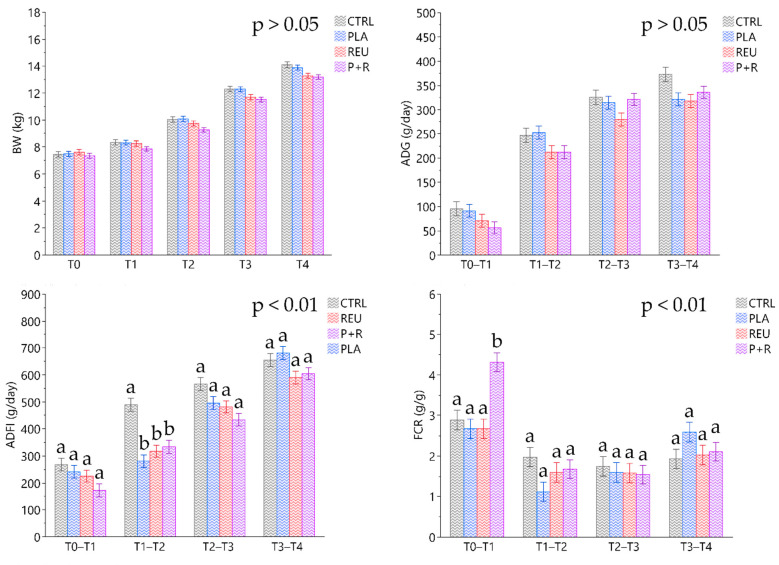
Zootechnical performance of control (CTRL) and treatment groups (PLA, REU and P+R) measured over 28 days of experimental trial. Data are expressed as least square means (LSMEANS) and standard errors of the means (SE); ^a,b^ Means with different superscripts are significantly different among treatments (*p* < 0.01); Presented *p*-values indicate statistically significances of pairwise comparisons; BW: body weight; ADG: average daily gain; ADFI: average daily feed intake; FRC: feed conversion ratio; CTRL: control group; PLA: treatment group supplemented with 2 × 10^8^ CFU/g of *L. plantarum*; REU: treatment group supplemented with 2 × 10^8^ CFU/g of *L. reuteri*; P+R: treatment group supplemented with 2 × 10^8^ CFU/g of *L. plantarum* and *L. reuteri* (1:1 *w*/*w*).

The results of individual BW recorded weekly showed no significant differences throughout the experimental period (Figure 3). The average BW of CTRL and PLA groups revealed a statistically significant tendency compared to P+R considering the entire experimental period (10.45 ± 0.19; 10.42 ± 0.17; 9.84 ± 0.16 kg, respectively; *p* < 0.09). In addition, the effect of treatments on ADG for the entire experimental period was significantly different for CTRL, REU and P+R groups, which showed a reduced average gain for treated groups with *L. reuteri* (CTRL: 260 ± 9, REU: 220 ± 8, P+R: 229 ± 7 g/day; *p* < 0.05). The ADFI of the supplemented groups decreased during the second week (7–14 days) of the study (CTRL: 490 ± 24, PLA: 281 ± 24; REU: 334 ± 24, P+R: 318 ± 22 g/day; *p* < 0.01). The FCR parameter highlighted an increased ratio in P+R group compared to CTRL, PLA and REU during the first week (0–7 days; CTRL: 2.89 ± 0.24; PLA: 2.67 ± 0.24; REU: 2.67 ± 0.24; P+R: 4.32 ± 0.23; *p* < 0.01).

### 3.8. Diarrhoea Occurrence

**Figure 4:**
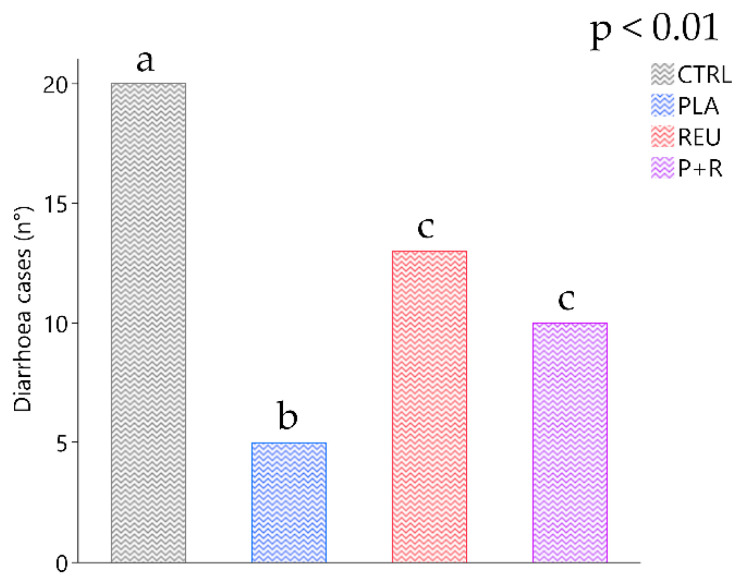
Total diarrhoea cases recorded during the 28-day trial for the control (CTRL) and treatment groups (PLA, REU and P+R). Data are expressed as the sum of recorded cases of diarrhoea, considering a faecal score ≥ 2 diarrhoea; ^a,b,c^ Means with different superscripts are significantly different among treatments (*p* < 0.01). CTRL: control group; PLA: treatment group supplemented with 2 × 10^8^ CFU/g of *L. plantarum*; REU: treatment group supplemented with 2 × 10^8^ CFU/g of *L. reuteri;* P+R: treatment group supplemented with 2 × 10^8^ CFU/g of *L. plantarum* and *L. reuteri* (1:1, *w*/*w*).

**Figure 5:**
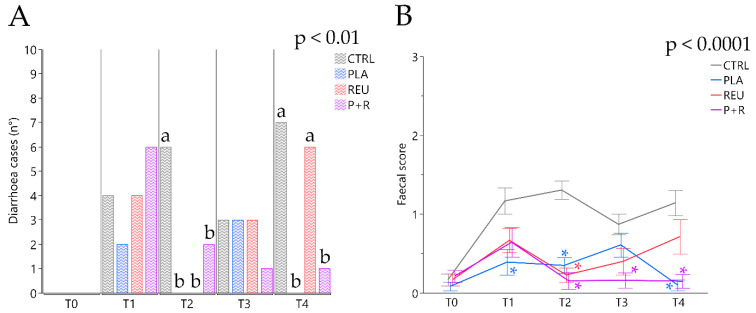
Number of diarrhoea cases recorded (**A**) and average faecal scores (**B**) during the 28-day trial for the control (CTRL) and treatment groups (PLA, REU and P+R). (**A**) Data are expressed as the sum of the recorded cases of diarrhoea, considering faecal score ≥ 2 diarrhoea; ^a,b^ Means with different superscripts are significantly different among treatments (*p* < 0.01). (**B**) Data are expressed as least square means (LSMEANS) and standard errors (SE); * Means with asterisks are significantly different from the control group (CTRL, *p* < 0.0001). CTRL: control group; PLA: treatment group supplemented with 2 × 10^8^ CFU/g of *L. plantarum*; REU: treatment group supplemented with 2 × 10^8^ CFU/g of *L. reuteri*; P+R: treatment group supplemented with 2 × 10^8^ CFU/g of *L. plantarum* and *L. reuteri* (1:1, *w*/*w*).

Considering the entire experimental period, diarrhoea observed frequencies differed significantly among treatments (*p* < 0.01). The highest number of cases of diarrhoea (20 cases) was found in the CTRL group, while 13 and 10 cases were recorded in the REU and P+R groups, respectively. The lowest number of diarrhoea cases (five cases) was recorded in the PLA group (Figure 4). Data on diarrhoea incidence considering each timepoint showed a statistically significant increase in CTRL compared to the treated groups at T2 (CTRL: 6 cases, 25.00%; PLA: 0 cases, 0.00%; REU 0 cases, 0.00%; P+R: 2 cases; 7.14%; *p* < 0.01) (Figure 5A). At the last sampling point (T4), diarrhoea occurrence was significantly lower in the PLA and P+R groups (CTRL: 7 cases; 29.17%; PLA: 0 cases, 0.00%; REU: 6 cases, 25.00%; P+R: 1 case; 3.57%; *p* < 0.01). Average faecal scores of representative subgroups of evaluated piglets revealed a higher score for the CTRL group compared with PLA at T1 (CTRL: 1.17 ± 0.13; PLA: 0.40 ± 0.13; *p* < 0.01) (Figure 5B). The average faecal score of CTRL after 14 days (T2) increased significantly compared with the treatment groups (CTRL: 1.31 ± 0.13; PLA: 0.34 ± 0.13; REU: 0.24 ± 0.13; P+R: 0.16 ± 0.12; *p* < 0.0001). At 21 days (T3), P+R highlighted a lower score compared to the CTRL group (CTRL: 0.89 ± 0.13; P+R: 0.18 ± 0.13; *p* < 0.05). PLA and P+R groups showed a significant decrease in average faecal score at the end of the trial compared to the CTRL group (CTRL: 1.16 ± 0.14; PLA: 0.13 ± 0.14; P+R: 0.17 ± 0.12; *p* < 0.0001).

### 3.9. Microbiological Analysis and Faecal pH

**Figure 6:**
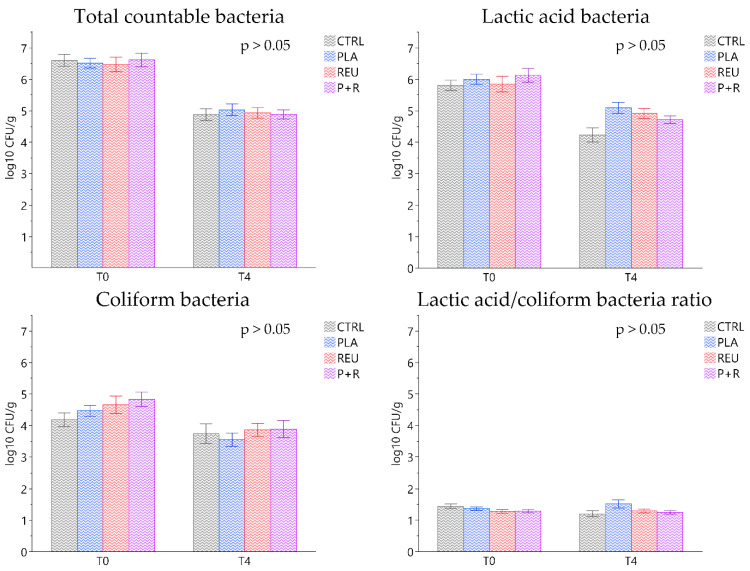
Faecal colonies of the principal bacterial groups (total countable bacteria, lactic acid bacteria, coliform bacteria and lactic acid/coliform ratio) for the control (CTRL) and treatment groups (PLA, REU and P+R) measured at the beginning (T0) and after 28 days of the trial (T4). Data are expressed as least square means (LSMEANS) and standard errors (SE). CTRL: control group; PLA: treatment group supplemented with 2 × 10^8^ CFU/g of *L. plantarum*; REU: treatment group supplemented with 2 × 10^8^ CFU/g of *L. reuteri;* P+R: treatment group supplemented with 2 × 10^8^ CFU/g of *L. plantarum* and *L. reuteri* (1:1, *w*/*w*).

Bacterial plate count results revealed no statistically significant difference among experimental groups at day 0 (T0) and after 28 days of the trial (T4) (Figure 6). However, a statistical tendency was observed for the lactic acid/coliform bacteria ratio at T4 comparing the CTRL and PLA groups (1.08 ± 0.10 and 1.54 ± 0.08 CFU/g, respectively; *p* < 0.09). Faecal pH measured at T4 revealed comparable averages among CTRL and treated groups (CTRL: 7.00 ± 0.07; PLA: 7.02 ± 0.08; REU: 7.24 ± 0.10; P+R: 7.09 ± 0.08).

### 3.10. Serum Metabolism

The results of serum metabolites showed no statistically significant differences over time for all experimental groups at T0 (Table S5). After 28 days, the PLA group showed a statistically significant increase in globulin content compared to the other groups (Table 3; *p* < 0.05). Consequently, the albumin/globulin ratio of the PLA group was lower than the other experimental groups (*p* < 0.05). Alanine aminotransferase (ALT) decreased significantly in the PLA and REU groups compared to the other groups (*p* < 0.01). The phosphorous concentration was higher in the P+R compared to PLA and REU groups (*p* < 0.05). The PLA group showed a decreased magnesium content in serum compared to the other groups (*p* < 0.05). Total cholesterol was lower in PLA and REU compared to the other experimental treatments (*p* < 0.05). In fact, high density lipoproteins were lower in PLA and REU compared to CTRL and P+R treatments (*p* < 0.01).

## 4. Discussion

Weaning is a critical phase characterised by a high incidence of gastrointestinal disorders. Probiotics may support intestinal health during this particular phase. This study focused on the effects of the dietary supplementation of *L. plantarum* and *L. reuteri* and their combination on the performance, metabolic status and gut health in weaned piglets. Lactic acid bacteria need to be ingested when administered as probiotics. They therefore need to reach the intestinal environment in a viable state in order to exert their wide range of positive activities. Probiotic bacteria are thus required to pass through the gastric environment where the pH reaches 2.5 [43]. 

The results related to acid tolerance showed that both *L. plantarum* and *L. reuteri* tolerate pH levels above 2 without losing significant viability. In line with our study, Yun et al. [44] demonstrated that *L. plantarum* and *L. reuteri* were able to resist pH levels from 4 to 9, meanwhile at pH 2, both strains showed a similar survival reduction (20%) after 6 h of incubation. Lukacova et al. [45] reported that more than 90% of *L. plantarum* strains need to survive at pH 3 in order to act as probiotics. 

In vitro simulated gastrointestinal tract transit tolerance is an important assay to evaluate the properties of probiotics. *L. plantarum* and *L. reuteri* strains showed an efficient ability to survive under each tested condition. In fact, *L. plantarum* and *L. reuteri* tolerate the gastric and small intestinal environment depending on the strain tested [38,46,47]. Bove et al. [48] evaluated the survival ability of *L. plantarum* WCFS1 in an oro-gastric-intestinal tract model, highlighting that this particular strain survives the entire digestion process. Our results suggest that *L. plantarum* and *L. reuteri* strains could be provided in feed and reach the intestinal environment without a significant viability loss, also without the need for other protection forms (e.g., microencapsulation).

The nutrient profile of experimental diets assessed by our chemical analyses was in line with post weaning piglet requirements following NRC guidelines [40]. Our experimental design considered a three-day adaptation period in order to enable piglets to adapt to the new environment and to be accustomed to feeding only on a solid diet, thus overcoming post-weaning fasting. Zootechnical performance is key to farm profitability and also an indirect index of animal health. Body weight showed a constant increase over the 28 days of our trial without significant differences among groups. Although the daily gain calculated for the 28 days was lower for REU and P+R than for CTRL, there were no differences considering all experimental groups for each timepoint (T1-T2-T3-T4). This thus highlighted that this slight reduction did not significantly impact the final body weight, ADG, ADFI and FCR of the animals. Other studies have shown a significant increase in body weight and average daily gain by including *L. plantarum* in the pig diet [25]. *L plantarum* supplemented at 10^9^ CFU/d showed an improved weight gain when administered for 60 days [49]. Bentancur et al. [50] orally administered 10^9^ CFU of *L. plantarum* CAM-6 from 21 to 49 days of age and found an increased daily gain and no differences in feed intake. In addition, *L. reuteri* strains supplementation have been shown to improve animal performance in weaned piglets [29,51]. Wang et al. [52] observed an increased feed intake and average daily gain with high doses of *L. reuteri* X-1 (10^11^ CFU/kg). Although several studies have shown boosting activity related to animal performance, the results are not directly comparable due to the different bacterial genotypes tested, animal ages, particular probiotic combinations, different in-feed inclusion levels or disparate supplementation methods. Furthermore, in line with EFSA guidelines [53], zootechnical performance effects should be better clarified with a long-term study supplementing probiotics at higher dosages (1 × 10^9^ CFU/g).

Gastrointestinal disorders are a major problem in swine farming during the weaning phase, when diarrhoea is one of the most evident dysbiosis signs and one of the principal reasons for prescribing antibiotics. On the other hand, eubiosis represents a healthy gut that is achieved through a positive interaction between the host, microorganisms and the environment. Our results revealed a lower diarrhoea occurrence in the lactobacilli-supplemented groups. The lowest diarrhoea cases were recorded in the *L. plantarum*-supplemented group (PLA). Over time, the lactobacilli-supplemented groups showed a lower faecal score, indicating an improved faecal consistency. The positive effect on diarrhoea was observed by administering different *L. plantarum* and *L. reuteri* strains to piglets individually. The prevention effects of *L. plantarum* on ETEC K88 have been observed in vitro and in vivo in a pig model through the stimulation of claudin-1, zonula occludens (ZO-1) and occludin expression, preventing epithelial barrier disruption [25,54]. Our results are in line with other studies where *L. reuteri* decreased diarrhoea incidence in piglets. In fact, *L. reuteri* supplemented at 2.4 × 10^5^ CFU/g as a lactobacilli preparation complex decreased diarrhoea incidence by over 60% [55]. The in-feed supplementation of *L. plantarum* and *L. reuteri* confirms their positive contribution to eubiosis in the intestinal environment. In addition, these results suggest that single strains or a combination of these lactic acid bacteria could help prevent diarrhoea. 

The bacterial plate count after weaning (T0) highlighted a high prevalence of lactic acid bacteria, and a reduction in this class was observed after 28 days (T4). In general, during the neonatal phase, lactobacilli and lactic acid bacteria are more common in piglets due to the consumption of maternal milk. They decrease in the post weaning phase frequently due to solid diet feeding [56]. During the trial, statistical differences in faecal viable bacterial counts were not detected among treatments and the control group. However, De Angelis et al. [57] found high viable lactobacilli in piglet faeces when 1 × 10^10^ CFU/pig *L. plantarum* 4.1 and *L. reuteri* 3S7 were administered for 15 days. The lactic acid/coliform bacteria ratio can be considered as a practical index for efficacy tests of feed additives, aimed at promoting the immune defence. Higher values of the lactobacilli:coliform ratio are normally associated with increased resistance to intestinal disorders [58]. Even only a statistical tendency of PLA group was registered compared to CTRL for this index at T4. The whole lactobacilli supplemented groups showed a lactic acid/coliform bacteria ratio above 1.3. On the other hand, CTRL showed a similar prevalence of lactic acid and coliform bacteria, suggesting that lactic acid was more predominant than coliform bacteria in supplemented groups.

The serum metabolic profile was useful in evaluating animals’ health and nutritional status, in order to clarify the possible interaction between bacteria and the host metabolism. Our results revealed that individual lactobacilli and their combined supplementation was safe without impairing animal metabolism, since all the values are in the normal range for pigs. The metabolic parameters showed higher levels of globulin which directly reduced the A/G ratio in PLA compared to the CTRL group. Globulins are mainly represented by immunoglobulins and are an important marker of immune system activity. Our results are in line with Dong et al. [59] who found a significant increase in globulin, with a simultaneous decrease in the A/G ratio after five weeks of *L. plantarum* GF103 supplementation. In addition, an increased concentration of IgA was observed by combining *L. plantarum* GF103 and *Bacillus subtilis* B27 [59]. The administration of microencapsulated *L. plantarum* and fructooligosaccharide blend has been found to increase plasma IgA and IgG concentrations in pigs [60]. Naqid et al. [61] observed that *L. plantarum* B2984 and lactulose dietary supplemented enhanced IgG production in response to *Salmonella typhimurium* infection in pigs. ALT can be exploited as a serum marker of liver damage, whose increase is related to cell membrane damage. Alanine aminotransferase is specific for liver tissues and is more effective in assessing a decrease in cell liver damage [62]. Although our results are in line with normal range for pigs [63,64,65,66], PLA and REU groups showed a significant reduction in serum ALT at 28 days suggesting a possible protective effect of *L. plantarum* and *L. reuteri* on liver cells. Fang et al. [32] showed that *L. plantarum* CMU995 supplementation decreased the ALT levels inhibiting alcohol-induced hepatitis. In line with our results, many probiotic species (*L. acidophilus*, *L. bulgaricus*, *Bifidobacterium lactis*, *Streptococcus termophylus*) demonstrated a protective effect on liver [67]. Phosphorous and magnesium are fundamental coenzymes and regulate many biochemical reactions in mammals. The P+R group had a higher P content after 28 days of supplementation with the lactobacilli combination. The magnesium serum concentration was lower in the PLA group than in other treatments. The interaction of dietary nutrients and the activity of microbiota are directly involved in mineral absorption [68]. P+R and PLA groups serum mineral concentrations suggest that *L. plantarum* administration could modulate mineral utilisation. Total cholesterol serum level is an index of the lipometabolic status, which includes the free and bounded forms of HDL [69]. The PLA and REU groups showed a significant reduction in total cholesterol, mainly due to the registered decrease of HDL concentration. *L. plantarum* and *L. reuteri* have been reported as positively contributing to cardiovascular diseases [70,71]. Certain probiotic strains could enhance faecal excretion of bile acids and resulting in a decrease of serum cholesterol concentration [72]. In line with this, *L. plantarum* 9-41-A significantly decreased hepatic cholesterol and TG levels when administered to rats fed a high-cholesterol diet [73]. Our results are likely due to the ability of the lactobacilli strain to modulate lipid metabolism, thereby preventing hypercholesterolemia. 

## 5. Conclusions

Dietary supplementation of 2 × 10^8^ CFU/g of *L. plantarum* and *L. reuteri* significantly reduced diarrhoea occurrence registering and had the lowest faecal score in our trial. *L. plantarum* had the lowest diarrhoea frequency compared to the other bacterial strains and their combinations. Lactobacilli supplementation did not influence animal performance, total faecal bacteria, faecal lactobacilli and coliform. Dietary lactobacilli inclusion did not reveal metabolic status alteration ascribable to a pathological status. In particular, *L. plantarum* significantly raised the globulin levels, suggesting a possible stimulation of the immune system. In conclusion, we believe that *L. plantarum* and *L. reuteri* are promising functional feed additives for preventing pig diarrhoea. More studies are required to enrich knowledge of these bacterial strains, to assess their effect for longer experimental periods, and to optimise their possible delivery systems.

## Figures and Tables

**Table 1:** Diet composition and principal chemical characteristics of experimental trial (% as fed basis) divided by control (CTRL, fed basal diet) and treatment groups (TRT, fed basal diet supplemented with 2 × 10^8^ CFU/g of *Lactobacillus plantarum*; 2 × 10^8^ CFU/g of *Lactobacillus reuteri* and 1 × 10^8^ CFU/g of both bacterial strains; PLA, REU and P+R, respectively).

Ingredients, % as Fed Basis		CTRL	TRT
Barley, meal		26.84	26.84
Wheat, meal		12.45	10.45
Corn, flakes		11.63	11.63
Corn, meal		10.00	10.00
Barley, flakes		7.50	7.50
Soy protein concentrates		5.00	5.00
Biscuits, meal		4.00	4.00
Soybean, meal		4.00	4.00
Dextrose monohydrate		3.50	3.50
Sweet milk whey		3.20	3.20
Herring, meal		2.00	2.00
Plasma, meal		2.00	2.00
Organic acids ^1^		1.70	1.70
Coconut oil		1.00	1.00
Soy oil		1.00	1.00
Arbocel ^2^		0.70	0.70
Dicalcium phosphate		0.60	0.60
L-Lysine		0.60	0.60
Benzoic acid		0.50	0.50
Vitamin and mineral premix ^3^		0.50	0.50
DL-Methionine		0.39	0.39
L-Threonine		0.35	0.35
Sodium Chloride		0.27	0.27
L-Valine (96.5%)		0.12	0.12
Enzymes ^4^		0.10	0.10
L-Tryptophan		0.05	0.05
Experimental mix ^5^		-	2.00
**Calculated Chemical Composition ^6^**			
Crude protein (%)	17.00		17.00
Fat (%)	4.20		4.20
Crude fibre (%)	2.90		2.90
Ashes (%)	5.20		5.20
DE ^7^ (Mc/Kg)	3.92		3.83

^1^ Citric acid, fumaric acid, orthophosphoric acid, sorbic acid, calcium formate. ^2^ Crude fibre concentrate (Rettenmaier & Söhne GmbH + Co KG, Rosenberg, Germany). ^3^ Additives per Kg: Vitamins, pro-vitamins and substances with similar effect. Retinyl Acetate 15,000 IU, Vitamin D3-Cholecalciferol 2000 IU, Vitamin E 120 mg, Vitamin B1 2.0 mg, Vitamin B2 4.8 mg, Vitamin B6 3.4 mg, Calcium D-pantothenate 15.0 mg, Vitamin B12 0.030 mg, Vitamin K3 1.9 mg, Biotin 0.19 mg, Niacinamide 30.0 mg, Folic Acid 0.96 mg, Vitamin C 144 mg, Choline chloride 288 mg, Betaine hydrochloride 1000 mg, Compounds of trace elements Iron sulphate 115 mg, Manganese Oxide 48.0 mg, Zinc Oxide 96.1 mg, Copper Oxide 130 mg, Anhydrous Calcium Iodate 0.96 mg, Sodium Selenite 0.34 mg. ^4^ 6-phytase, endo-1,4-beta-xylanase, endo-1,3(4)-beta-glucanase. ^5^ Experimental mix was composed of wheat flour 00, and the respective bacterial strain according to dietary treatments: *L. plantarum* (PLA), *L. reuteri* (REU), *L. plantarum* and *L. reuteri* combination (P+R) in order to reach a final concentration of 2 × 108 CFU/g in the complete diet. ^6^ Calculation performed with Purimix System^®^ software (Fabermatica, Cremona, Italy). ^7^ DE: digestible energy content estimated from NRC (2012).

**Table 2:** Chemical composition of experimental diets divided by control (CTRL) and treatment groups (PLA, REU and P+R).

**Analyte**	**CTRL**	**PLA**	**REU**	**P+R**
DM	90.89	91.14	91.14	90.78
CP	16.34	17.01	16.38	16.64
EE	3.98	3.78	3.74	3.80
CF	3.60	3.65	3.34	3.40
Ashes	4.59	4.49	4.54	4.25
**FA Composition (% Total FAMEs)**	**CTRL**		**TRT**	
Caproic acid, C6:0	0.04		0.04	
Caprylic acid, C8:0	1.00		1.10	
Capric acid, C10:0	1.12		1.21	
Undecanoic acid, C11:0	0.00		0.00	
Lauric acid, C12:0	10.85		11.69	
Tridecanoic acid, C13:0	0.01		0.01	
Myristic acid, C14:0	5.23		5.45	
Mysticoleic acid, C14:1	0.01		0.01	
Pentadecanoic acid, C 15:0	0.05		0.06	
cis-10 Heptadecenoic acid, C 17:0	0.00		0.00	
Stearic acid, C 18:0	15.17		14.95	
Elaidic acid, C 18:1 n9t	0.23		0.22	
Oleic acid, C 18:1 n9c	0.09		0.08	
Linolelaidic acid, C18:2 n6t	0.00		0.00	
Linoleic acid, C 18:2 n6c	4.34		4.07	
γ-Linolenic acid, C 18:3 n6	0.05		0.04	
α-Linolenic acid, C18:3 n3	22.71		22.50	
Arachidic acid, C 20:0	0.00		0.00	
Cis-11 Eicosenoic acid, C20:1	34.42		33.92	
Cis-11,14 Eicosenoic acid, C20:2	0.04		0.04	
Cis-8,11,14 Eicosatrienoic acid, C20:3 n6	2.65		2.74	
Cis-11,14,17 Eicosatrienoic acid, C20:3 n3	0.31		0.29	
Arachidonic acid, C20:4 n6	0.43		0.40	
Cis-5,8,11,14,17 Eicosapentaenoic acid, C20:5 n3	0.05		0.04	
Heneicosanoic acid, C21:0	0.00		0.00	
Behenic acid, C22:0	0.01		0.01	
Erucic acid, C22:1 n9	0.02		0.02	
Cis-13,16 Docosadienoic acid, C22:2	0.26		0.25	
Cis-4,7,10,13,16,19 Docosahexaenoic acid, C22:6 n3	0.02		0.02	
Lignoceric acid; C24:0	0.25		0.23	
Nervonic acid, C24:1	0.04		0.04	
SFA	38.64		39.36	
MUFA	23.52		23.25	
PUFA	37.83		37.39	

DM: dry matter; CP: crude protein; EE: ether extract; CF: crude fibre; FA: fatty acids; FAMEs: fatty acid methyl esters; SFA: saturated fatty acids, MUFA monounsaturated fatty acids, PUFA: polyunsaturated fatty acids. All values are expressed as percentage as fed basis (%). CTRL: control group; PLA: treatment group supplemented with 2 × 10^8^ CFU/g of *L. plantarum*; REU: treatment group supplemented with 2 × 10^8^ CFU/g of *L. reuteri;* P+R: treatment group supplemented with 2 × 10^8^ CFU/g of *L. plantarum* and *L. reuteri* (1:1 *w*/*w*); TRT: treatment group supplemented with 2 × 10^8^ CFU/g of lactobacilli.

**Table 3:** Serum metabolites concentration at 28 days (T4) of in vivo trial, for the control (CTRL) and treatments groups (PLA, REU and P+R).

Serum Metabolite	CTRL	PLA	REU	P+R	*p*-Value
Total protein content, g/L	53.26 ± 1.23	54.85 ± 1.15	51.84 ± 1.15	52.09 ± 1.15	0.2576
Albumin, g/L	28.35 ± 0.74	26.00 ± 0.70	25.94 ± 0.70	26.77 ± 0.70	0.0916
Globulin, g/L	24.91 ± 1.09 ^a^	28.89 ± 1.03 ^b^	25.91 ± 1.03 ^a^	25.31 ± 1.03 ^a^	0.0455
Albumin/Globulin (A/G)	1.16 ± 0.05 ^a^	0.92 ± 0.05 ^b^	1.06 ± 0.05 ^a^^,^^b^	1.01 ± 0.05 ^a,b^	0.0287
Urea, mmol/L	1.06 ± 0.21	1.42 ± 0.20	0.96 ± 0.20	0.89 ± 0.20	0.2452
Alanine aminotransferase (ALT-GPT), IU/L	50.00 ± 2.89 ^a^	38.22 ± 2.73 ^b^	35.78 ± 2.73 ^b^	46.00 ± 2.73 ^a,b^	0.0034
Total bilirubin, µmol/L	1.84 ± 0.13	1.42 ± 0.12	1.45 ± 0.12	1.58 ± 0.12	0.1021
Glucose, mmol/L	6.36 ± 0.47	6.31 ± 0.44	5.36 ± 0.44	6.41 ± 0.44	0.2926
Phosphorus, mmol/L	3.19 ± 0.09 ^a,b^	2.87 ± 0.08 ^a^	2.98 ± 0.08 ^a^	3.30 ± 0.08 ^b^	0.0038
Magnesium, mmol/L	0.92 ± 0.04 ^a^	0.77 ± 0.11 ^b^	0.79 ± 0.12 ^a,b^	0.85 ± 0.13 ^a,b^	0.0196
Creatinine, µmol/L	70.75 ± 3.51	77.33 ± 3.31	76.00 ± 3.31	81.10 ± 3.31	0.2175
Total cholesterol, mmol/L	2.70 ± 0.14 ^a^	2.20 ± 0.13 ^b^	2.27 ± 0.13 ^b^	2.68 ± 0.13 ^a^	0.0195
High density lipoprotein (HDL), mmol/L	1.08 ± 0.06 ^a^	0.77 ± 0.06 ^b^	0.81 ± 0.06 ^b^	1.04 ± 0.06 ^a^	0.0011
Low density lipoprotein (LDL), mmol/L	1.50 ± 0.09	1.26 ± 0.09	1.31 ± 0.09	1.52 ± 0.09	0.1173
Triglycerides, mmol/L	0.58 ± 0.08	0.83 ± 0.07	0.70 ± 007	0.60 ± 0.07	0.0764
Interleukin 3, pg/L	17.80 ± 1.98	14.78 ± 2.17	17.28 ± 2.17	17.90 ± 2.17	0.7098
Interleukin 6, pg/L	166.47 ± 45.87	152.65 ± 45.87	155.06 ± 45.87	166.48 ± 45.87	0.9941
Interleukin 10, pg/L	10.67 ± 2.13	8.91 ± 2.13	8.49 ± 2.13	10.80 ± 2.13	0.8158

Data are expressed as least square means (LSMEANS) ± standard errors (SE). ^a,b^ Means with different superscripts are significantly different among treatments (*p* < 0.05). CTRL: control group; PLA: treatment group supplemented with 2 × 10^8^ CFU/g of *L. plantarum*; REU: treatment group supplemented with 2 × 10^8^ CFU/g of *L. reuteri;* P+R: treatment group supplemented with 2 × 10^8^ CFU/g of *L. plantarum* and *L. reuteri* (1:1, *w*/*w*).

## Data Availability

The data presented in this study are available within the article and supplementary materials.

## Supplementary Materials

The following are available online at https://www.mdpi.com/article/10.3390/ani11061766/s1, Figure S1: Agarose gel electrophoresis of *L. plantarum* PCR reaction products, Figure S2: Agarose gel electrophoresis of *L. reuteri* PCR reaction products, Table S1: MIC concentrations (µg/mL) obtained for *L. plantarum* and *L. reuteri* strains and cut-off values proposed from EFSA guidance on the assessment of bacterial susceptibility to antimicrobials of human and veterinary importance, Table S2: Obtained biomass from small-scale fermentations and viability of *L. plantarum* and *L. reuteri,* Table S3: Obtained biomass and relative viability of freeze-dried *L. reuteri* and *L. plantarum*, Table S4: Bacterial fermentation for experimental trial batch production, Table S5: Serum metabolites concentration at 0 days (T0) of in vivo trial, for the control (CTRL) and treatments groups (PLA, REU and P+R).
