# Supplementary material for: Lactobacillus plantarum and Lactobacillus reuteri as Functional Feed Additives to Prevent Diarrhoea in Weaned Piglets
Source: Animals (Basel). 2021 Jun 12;11(6):1766. doi: 10.3390/ani11061766 (PMC8231520; doi:10.3390/ani11061766)
Supplement: Supplementary file 1 [file animals-11-01766-s001.zip › animals-1236417-supplementary.pdf]

Supplementary materials

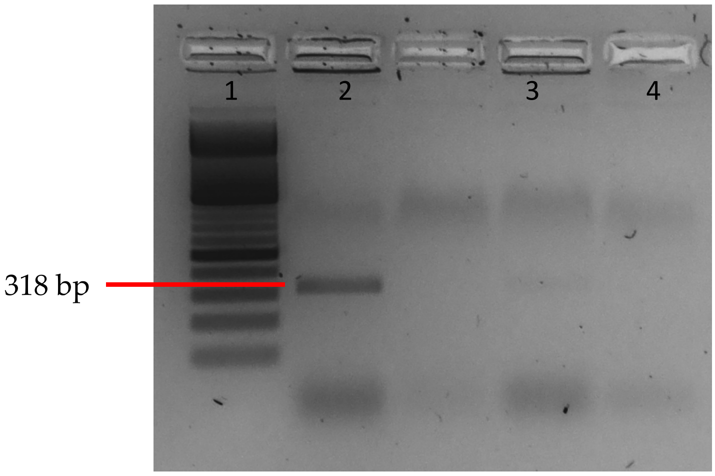

Figure S1: Agarose gel electrophoresis of *L. plantarum* PCR reaction products.
Molecular weight marker (1); sample DNA: *L. plantarum* (2); positive control DNA: *L. plantarum* ATCC®14917™ (3); negative control (4).

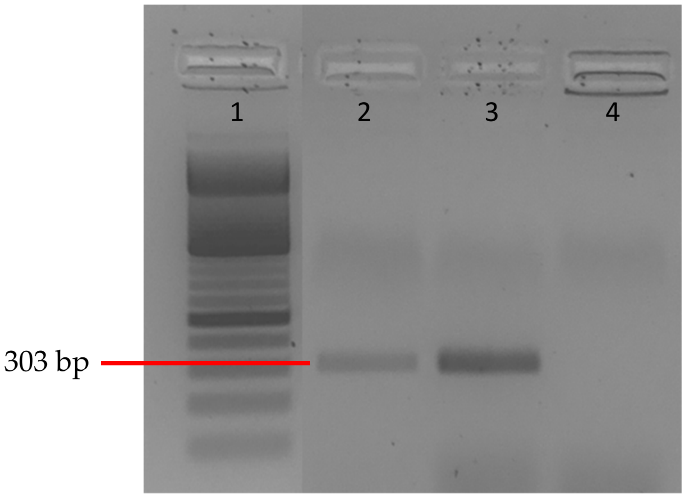

Figure S2: Agarose gel electrophoresis of *L. reuteri* PCR reaction products.
Molecular weight marker (1); sample DNA: *L. reuteri* (2); positive control DNA: *L. reuteri* DSM 20016 (3); negative control (4).

Table S1. MIC concentrations (µg/mL) obtained for *L. plantarum* and *L. reuteri* strains and cut-off values proposed from EFSA guidance on the assessment of bacterial susceptibility to antimicrobials of human and veterinary importance [1].

Antibiotic	*L. plantarum*	*L. reuteri*	EFSA Cut-off
Ampicillin	0.25	1.00	2.00
Chloramphenicol	8.00	8.00	8.00
Ciprofloxacin	32.00	8.00	n.l.
Clindamycin	8.00	1.00	2.00
Erythromycin	0.50	0.25	1.00
Gentamicin	8.00	1.00	16.00
Kanamycin	> 64.00	32.00	64.00
Linezolid	4.00	4.00	n.l.
Neomycin	16.00	2.00	n.l.
Penicillin	0.50	2.00	n.l.
Quinupristin/Dalfopristin	4.00	2.00	n.l.
Rifampicin	1.00	< 0.12	n.l.
Streptomycin	64.00	16.00	64.00*
Tetracycline	16.00	32.00	32.00
Trimethoprim	> 64.00	> 64	n.l.
Vancomycin	> 128.00	> 128	n.r.

n.l. = not listed value

n.r. = not required value

*Cut-off value listed only for *L. reuteri*, the following value is not requested for *L. plantarum*.

Table S2. Obtained biomass from small-scale fermentations and viability of *L. plantarum* and *L. reuteri*.

	L. plantarum		*L reuteri*	
Fermentation time (h)	Biomass (g)	CFU/g	Biomass (g)	CFU/g
18	39.12	2.52×10^{11}	29.71	1.10×10^{11}
24	40.07	2.25×10^{11}	27.63	1.72×10^{11}

Table S3. Obtained biomass and relative viability of freeze-dried *L. reuteri* and *L. plantarum.*

Bacterial strain	Biomass (g)	Freeze-dried bacteria (g)	CFU/g biomass	CFU/g of freeze-dried bacteria
L. plantarum	90.82	14.83	1.45×10^{13}	8.63×10^{12}
L. reuteri	56.28	9.17	4.24×10^{12}	2.23×10^{12}

Table S4. Bacterial fermentation for experimental trial batch production**.**

Bacterial strain	Biomass (g)	Freeze-dried bacteria (g)	CFU/g biomass	Total CFU of freeze-dried bacteria
L. plantarum	206.15	32.61	1.71×10^{13}	9.39×10^{11}
L. reuteri	376.46	68.22	3.95×10^{13}	1.26×10^{13}

Table S5. Serum metabolites concentration at 0 days (T0) of *in vivo* trial, for the control (CTRL) and treatments groups (PLA, REU and P+R).

Serum metabolite	CTRL	PLA	REU	P+R	*P*-Value
Total protein content, g/L	53.82 ± 3.10	47.70 ± 6.94	54.10 ± 6.94	49.83 ± 4.01	0.7778
Albumin, g/L	33.46 ± 1.30	29.4 ± 2.90	28.9 ± 2.90	31.07 ± 1.68	0.3972
Globulin, g/L	20.32 ± 2.22	18.30 ± 4.96	25.10 ± 4.96	18.77 ± 2.86	0.7213
Albumin/Globulin (A/G)	1.70 ± 0.16	1.61 ± 0.35	1.15 ± 0.35	1.74 ± 0.20	0.5544
Urea, mmol/L	1.72 ± 0.73	0.90 ± 1.64	0.70 ± 1.64	1.70 ± 0.95	0.9142
Alanine aminotransferase (ALT-GPT), IU/L	35.00 ± 1.86	35.00 ± 4.16	24.00 ± 4.16	28.00 ± 2.40	0.1057
Total bilirubin, µmol/L	2.44 ± 0.25	2.00 ± 0.57	2.60 ± 0.57	2.03 ± 3.33	0.6924
Glucose, mmol/L	5.02 ± 0.48	5.50 ± 1.07	5.00 ± 1.07	4.97 ± 0.62	0.9757
Phosphorus, mmol/L	3.54 ± 0.16	3.70 ± 0.36	3.20 ± 0.36	3.27 ± 0.21	0.5949
Magnesium, mmol/L	1.14 ± 0.04	0.96 ± 0.08	1.08 ± 0.08	1.02 ± 0.05	0.2378
Creatinine, µmol/L	104.60 ± 5.81	80.00 ± 12.99	91.00 ± 12.99	100.33 ± 7.50	0.4015
Total cholesterol, mmol/L	2.28 ± 0.13	1.99 ± 0.30	2.49 ± 0.30	2.16 ± 0.17	0.665
High density lipoprotein (HDL), mmol/L	0.90 ± 0.07	0.90 ± 0.17	0.82 ± 0.17	0.86 ± 0.10	0.9685
Low density lipoprotein (LDL), mmol/L	1.24 ± 0.12	0.93 ± 0.26	1.41± 0.26	1.20 ± 0.15	0.6374
Triglycerides, mmol/L	0.73 ± 0.12	0.80 ± 0.27	1.30 ± 0.27	0.53 ± 0.15	0.1968
Interleukin 3, pg/L	14.87 ± 1.99	11.61 ± 2.30	13.04 ± 3.98	15.21 ± 2.30	0.6844
Interleukin 6, pg/L	138.05 ± 20.27	95.83 ± 23.40	142.22 ± 40.53	92.63 ± 23.40	0.4072
Interleukin 10, pg/L	9.57 ± 0.76	9.73 ± 0.88	7.73 ± 1.53	8.95 ± 0.88	0.6793

Data are expressed as least square means (LSMEANS) ± standard errors (SE).
CTRL: control group; PLA: treatment group supplemented with 2×10^8 CFU/g of *L. plantarum*; REU: treatment group supplemented with 2×10^8 CFU/g of *L. reuteri;* P+R: treatment group supplemented with 2×10^8 CFU/g of *L. plantarum* and *L. reuteri* (1:1, w/w).

Reference

1. EFSA Panel on Additives and Products or Substances used in Animal Feed. Guidance on the assessment of bacterial susceptibility to antimicrobials of human and veterinary importance. *EFSA Journal* **2012**, *10*, 2740.
